# Enzybiotic-mediated antimicrobial functionalization of polyhydroxyalkanoates

**DOI:** 10.3389/fbioe.2023.1220336

**Published:** 2023-06-28

**Authors:** Francisco G. Blanco, Roberto Vázquez, Ana M. Hernández-Arriaga, Pedro García, M. Auxiliadora Prieto

**Affiliations:** ^1^ Polymer Biotechnology Group, Microbial and Plant Biotechnology Department, Margarita Salas Center for Biological Research (CIB–CSIC), Madrid, Spain; ^2^ Interdisciplinary Platform of Sustainable Plastics towards a Circular Economy, Spanish National Research Council (SusPlast-CSIC), Madrid, Spain; ^3^ Protein Engineering Against Antibiotic Resistance Group, Microbial and Plant Biotechnology Department, Margarita Salas Center for Biological Research (CIB-CSIC), Madrid, Spain

**Keywords:** polyhydroxyalkanoates, antimicrobial nanoparticles, antimicrobial materials, enzybiotics, drug delivery

## Abstract

Polymeric nanoparticles (NPs) present some ideal properties as biomedical nanocarriers for targeted drug delivery such as enhanced translocation through body barriers. Biopolymers, such as polyhydroxyalkanoates (PHAs) are gaining attention as nanocarrier biomaterials due to their inherent biocompatibility, biodegradability, and ability to be vehiculized through hydrophobic media, such as the lung surfactant (LS). Upon colonization of the lung alveoli, below the LS layer, *Streptococcus pneumoniae*, causes community-acquired pneumonia, a severe respiratory condition. In this work, we convert PHA NPs into an antimicrobial material by the immobilization of an enzybiotic, an antimicrobial enzyme, via a minimal PHA affinity tag. We first produced the fusion protein M711, comprising the minimized PHA affinity tag, MinP, and the enzybiotic Cpl-711, which specifically targets *S. pneumoniae*. Then, a PHA nanoparticulate suspension with adequate physicochemical properties for pulmonary delivery was formulated, and NPs were decorated with M711. Finally, we assessed the antipneumococcal activity of the nanosystem against planktonic and biofilm forms of *S. pneumoniae*. The resulting system displayed sustained antimicrobial activity against both, free and sessile cells, confirming that tag-mediated immobilization of enzybiotics on PHAs is a promising platform for bioactive antimicrobial functionalization.

## 1 Introduction

Nanocarriers, or colloidal systems for therapeutic applications, play a prominent role in current biomedical research. Their most common formulation is nanoparticulate suspensions, which, due to their nano size, effectively cross different body barriers, depending on their chemical nature (Jia et al., 2020). Ideally, these biomedical nanocarriers should be biocompatible, biodegradable, with optimal bioavailability, and display sustained bioactivity over time (Li and Loh, 2017). Polymers are materials of interest in the biomedical field due to the design flexibility based on their diversity in chemical nature, synthesis methods, and functionalization approaches (Banik et al., 2016). Although initial research focused on using chemically synthesized polymers such as poly (ε-caprolactone) or polylactic-*co*-glycolic acid (Cabeza et al., 2017; Amararathna et al., 2022), biopolymers have recently gained interest due to their inherent biocompatibility, biodegradability, and potentially sustainable production processes (Blanco et al., 2021).

Among them, polyhydroxyalkanoates (PHAs), or bacterial polyesters, have been studied for biomedical applications. They are polymers of 3-hydroxyalkanoic acids bearing a wide range of substituents, being the most common aliphatic groups (Mezzina et al., 2021). This confers the resulting polymers a relatively high hydrophobicity, which is advantageous for their vehiculization through challenging body barriers, such as the lung surfactant (Guagliardo et al., 2018; Cañadas et al., 2021). PHAs are naturally synthesized by bacteria as carbon reservoirs in the form of cytoplasmic granules (Kniewel et al., 2019). These granules are covered by a layer of granule-associated proteins (GAPs), mainly synthases, depolymerases, and phasins. The latter are surfactant proteins that coat the hydrophobic polymer, providing a compatible interface with the hydrophilic cytoplasm (Maestro and Sanz, 2017). Their surface-active properties have been extensively studied due to their ability to interact at hydrophilic-hydrophobic interfaces such as the air-water interface, the polymer-buffer interface, with bacterial lipid extracts, or with lung surfactant models (Tarazona et al., 2019a; Mato et al., 2019). These proteins, or their engineered versions, have been used as affinity tags towards PHA-based materials. For instance, a small peptide of 48 amino acids, MinP, was designed based on the binding moiety of phasin PhaF from *Pseudomonas putida.* MinP maintains the global binding capability of the whole protein (Mato et al., 2020). Phasin-mediated immobilization has been demonstrated both *in vivo*, where the PHA synthesis, protein production, and binding to the polymer take place simultaneously within the cytoplasm of the bacterial host; and *in vitro*, where proteins are immobilized onto previously formulated materials (Moldes et al., 2004; Bello-Gil et al., 2018; Mato et al., 2020). The *in vitro* approach is more suitable to ensure endotoxin removal from both the polymer and the protein when targeting biomedical applications (Dinjaski and Prieto, 2015), as well as to tightly control protein load. This approach becomes particularly challenging when the molecule to immobilize is an enzyme, which calls for orientated attachment to maintain its bioactivity. Some approaches based on other GAPs such as protein fusion with synthases and depolymerases have been developed for biomedical applications (Lee et al., 2005; Gonzalez-Miro et al., 2019). However, despite the advantages that phasin system provides, it has been scarcely applied to formulate antimicrobial materials: only one study has previously focused on phasin-mediated immobilization of antimicrobials on PHA by using phasin PhaP from *Cupriavidus necator* to immobilize an antimicrobial peptide onto a PHA polymer for wound healing (Xue et al., 2018).

Regarding antimicrobial applications, the rise of antibiotic resistance is one of the major challenges Global Health is facing in the next decades (Murray et al., 2022). This has fueled the development of new antimicrobial strategies to bypass antibiotic resistance (Rojo et al., 2022). One of such new approaches are “enzybiotics” or enzymatic antimicrobials. These antibacterial proteins are usually phage endolysins, peptidoglycan-degrading enzymes that, when exogenously applied against bacteria, produce a fast bacterial death due to cell wall disruption and subsequent osmotic shock (Murray et al., 2021). They present several advantages over antibiotics: i) activity against antibiotic-resistant strains and biofilms; ii) spontaneous resistance is less likely to occur than with antibiotics; and iii) their specificity is easily tunable by protein engineering, thereby reducing possible side effects on the healthy microbiota (Vázquez et al., 2018). However, their rather short *in vivo* half-life (20–60 min) may hamper their therapeutic use (Loeffler Jutta M. et al., 2003). Thus, the development of tailored formulations has become a hotspot to improve the therapeutic properties of this new kind of antimicrobials (De Maesschalck et al., 2020), as proved by the recent efforts towards the immobilization or encapsulation of enzybiotics (Miao et al., 2011; Nithya et al., 2018; Gondil et al., 2020; Urbanek et al., 2021; Vázquez et al., 2021). *Streptococcus pneumoniae* remains the main cause of bacterial respiratory infections in children and the elderly worldwide (Sempere et al., 2020). Its biofilm lifestyle, together with the high percentage of antibiotic-resistant strains and insufficient coverage of serotypes in vaccination, are some of the reasons behind its inclusion in the priority list for the development of antimicrobials elaborated by the World Health Organization (World Health Organization, 2017). Nevertheless, enzybiotics have proven to be a powerful tool to overcome antibiotic resistance on this pathogen (Vázquez et al., 2018).

Within this context, the aim of this work was to develop a platform for affinity immobilization of enzybiotics on PHA-based materials that allows the antimicrobial activity of the enzyme to be maintained. Thus, we fused the antipneumococcal lysin Cpl-711, a very efficient enzybiotic against this pathogen (Díez-Martínez et al., 2015), to the minimal PHA affinity tag MinP (Mato et al., 2020) and developed a procedure to immobilize such fusion protein (M711) onto preformed PHA NPs. Finally, as a proof of concept, we assessed the antipneumococcal potential of such functionalized material.

## 2 Materials and methods

### 2.1 Materials

The poly-3-hydroxyoctanoate-*co*-3-hydroxyhexanoate (hereinafter PHA) copolymer, with a respective monomer molar ratio of 94% and 6%, was kindly supplied by Bioplastech Ltd (Dublin, Ireland).

### 2.2 Bacterial strains, media, and growth conditions

The bacteria and plasmids used in this study are listed in Table 1. *P. putida* and *Escherichia coli* strains were grown on lysogeny broth (LB) medium at 30 or 37°C respectively, with shaking (200 rpm). Kanamycin (Km) or ampicillin (Amp) were added when needed at a final concentration of 50 μg mL^−1^ (Km) or 100 μg mL^−1^ (Amp). The PHA accumulation conditions for *P. putida* cultures were as described in (Mato et al., 2020). Briefly, the biomass from a 20 mL LB overnight culture was pelleted and washed with 120 mM NaCl, and then used to inoculate fresh 0.1 N M63 medium (with 15 mM sodium octanoate as carbon source) to an OD_600_ = 0.3 (Mato et al., 2020). *S. pneumoniae* R6 was grown at 37°C without shaking in Todd-Hewitt broth (Difco, NJ, United States of America) with 2% yeast extract. *S. pneumoniae* P046 for biofilm assays was grown in C medium (Lacks and Hotchkiss, 1960) containing 33 mM potassium phosphate buffer pH 8.0 (CpH8) at 37°C without shaking. Blood agar plates (trypticase soy agar plus 0.05% defibrinated sheep blood) were used to culture *S. pneumoniae* in solid medium. Solid media for *E. coli* and *P. putida* cultures were LB-agar plates containing the corresponding antibiotic, and incubated at 37°C or 30°C, respectively.

**TABLE 1:** Plasmids and bacterial strains used in this study.

Plasmid	Description	Plasmid
pTRD762	pUC derivative plasmid bearing the gene encoding Cpl-711 under the control of the T7 promoter. AmpR	Díez-Martínez et al. (2015)
pFB85	pBBR1 derivative plasmid bearing the gene encoding M711 under the control of the XylS/Pm system. KmR	This study

Strain	Description	Reference
*E. coli* BL21 (DE3) (pTRD762)	Strain for the production of Cpl-711	Díez-Martínez et al. (2015)
*E. coli* BL21 (DE3) (pFB85)	Strain for the production of M711	This study
*P. putida* PP00_01	*P. putida* KT2440 ∆*pha,* with PP_5003 (*phaC1*) integrated at the Tn7 site, which produces bare PHA granules, without the presence of GAPs	Mato et al. (2020)
*P. putida* PP00_01 (pFB85)	*P. putida* PP00_01 harboring the plasmid for M711 production, which produces M711 immobilized onto PHA granules	This study
*S. pneumoniae* R6	Laboratory standard strain. D39 derivative (serotype 2); Non-encapsulated	Hoskins et al. (2001)
*S. pneumoniae* P046	*S. pneumoniae* R6 derivative, *lytA::kan lytC::ermC*. Non autolytic strain of *S. pneumoniae*	Moscoso et al. (2006)

### 2.3 DNA techniques

The synthetic gene *m711*, comprising the MinP PHA affinity tag directly fused to Cpl-711 (Supplementary Figure S1A) (thus M711 chimeric protein) was purchased from ATG:Biosynthetics (Merzhausen, Germany), and cloned at the NdeI-HindIII restriction sites in a pSEVA238 expression vector (Silva-Rocha et al., 2013). The resulting vector (pFB85) was then transformed either by heat shock into *E. coli* BL21 (DE3) competent cells or by electroporation into *P. putida* PP00_01 cells according to the methods described previously (Mato et al., 2020).

### 2.4 Protein techniques

#### 2.4.1 Protein production

The production of Cpl-711 or M711 was carried out by culturing the corresponding *E. coli* or *P. putida* strain and inducing at an OD_600_ = 0.6 by the addition of 0.5 mM isopropyl-β-d-thiogalactopyranoside (IPTG) for Cpl-711, or 1 mM 3-methylbenzoate (3-MB) for M711. Incubation was then prolonged overnight at room temperature (RT). Cells were subsequently harvested by centrifugation (10,000 × *g*, 20 min, 4°C), resuspended in the binding buffer (20 mM sodium phosphate pH 6.0, 1.5 M NaCl) with two tablets of protease inhibitor (Roche, Switzerland) per 10 mL of resuspended culture, and disrupted three times in a French Press (at ≈ 1,000 psi). Cell lysates were clarified by centrifugation (10,000 × *g*, 20 min, 4°C) to remove cell debris, and the remaining DNA present in the lysate was further eliminated by precipitation with 4% (w/v) of streptomycin sulfate under magnetic stirring (200 rpm, 20 min, 4°C) and further centrifugation (10,000 × *g*, 20 min, 4°C).

When the production of M711 was done in *P. putida* PP00_01 (pFB85) under PHA-accumulation conditions, the procedure was the same but the binding buffer additionally contained 1.6 mM sodium dodecyl sulfate (SDS) and the cell lysates were incubated for 2 h at 30°C with shaking (200 rpm) to promote M711 detachment from the PHA granules.

#### 2.4.2 Protein purification

Proteins were purified based on Cpl-711 affinity towards the diethylaminoethyl (DEAE) group, a structural analogue to its natural ligand, choline (Sanz et al., 1988). The clarified cell extracts were loaded onto a DEAE-Sepharose column (HiTrap DEAE FF 5 mL, GE Healthcare, United States) equilibrated with binding buffer, then were washed with the same buffer and finally eluted with elution buffer (20 mM sodium phosphate pH 6.0, 1.5 M NaCl, 4% choline). The eluted protein fractions were analyzed by SDS-PAGE (12.5%) and dialyzed against 20 mM sodium phosphate buffer pH 6.0, 150 mM NaCl. This was also the buffer in which the antibacterial activity was tested (hereinafter “activity buffer”).

Protein concentrations were calculated from A_280_ with the molar extinction coefficient as predicted with ProtParam (Gasteiger et al., 2005) (126,865 M^−1^ cm^−1^ for Cpl-711, and 140,845 M^−1^ cm^−1^ for M711).

#### 2.4.3 N-terminal sequencing

To verify the identity of the purified proteins, N-terminal sequencing was performed. Samples from different M711 production culture conditions were subjected to SDS-PAGE (12.5%) and then transferred onto methanol activated - polyvinylidene fluoride (PVDF) membranes in a semidry transfer device (Biorad, CA, United States) soaked in transfer buffer (25 mM Tris, 192 mM glycine, 20% methanol, pH 8.3) for 1 h 15 min at 15 mV. The resulting transferred membranes were stained with Ponceau S stain (ThermoFisher, MA, United States), and the visible protein bands were subjected to N-terminal sequencing by Edman degradation (Edman and Begg, 1967) in a Protein sequencer (Applied Biosystems, Procise 494, CA, United States), as performed by the Protein Chemistry service from the Margarita Salas Center for Biological Research.

### 2.5 Preparation of PHA granules

The PHA granules from *P. putida* PP00_01 (pFB85) were isolated as previously described (Dinjaski and Prieto, 2013). Bacteria from 20 mL cultures grown overnight under PHA-accumulation conditions were harvested (12,000 x *g*, 20 min, 4°C), resuspended in 8 mL of 15 mM Tris-HCl pH 8.0, and disrupted twice in a French press (≈1,000 psi). The resulting suspension was centrifuged (12,000 x *g*, 30 min, 4°C) and the supernatant was layered over 5 mL of 55% glycerol. The suspension was centrifuged (18,000 x *g*, 30 min, 4°C), and the granules were isolated with a Pasteur pipette from the glycerol-buffer interface. Granules were washed twice with 15 mM Tris-HCl buffer, pH 8.0.

### 2.6 PHA nanoparticle procedures

#### 2.6.1 Nanoparticle formulation

PHA solid nanoparticles (PHA NPs) were produced by the nanoprecipitation method as previously reported (Beck-Broichsitter et al., 2010). 100 mg of PHA were dissolved in 50 mL of acetone, and the solution was added dropwise onto 10 mL of distilled water placed on ice water while magnetically stirred (250 rpm). The resulting suspension was vacuum evaporated at 65°C for 5 min to remove the acetone. The PHA NPs were then leveled to 10 mL of distilled water and stored as a 10 mg mL^−1^ PHA NPs stock solution at 4°C for up to 7 days.

#### 2.6.2 Loading M711 onto PHA NPS

To functionalize PHA NPs with M711, 100 µL of the 10 mg mL^−1^ NPs stock solution were centrifuged (13,000 x *g*, 30 min), resuspended in 100 µL of a 250 nM M711 solution in activity buffer, and incubated in an orbital shaker (800 rpm, 30 min). The resulting M711-NPs and protein mixture was centrifuged (13,000 x *g*, 30 min), washed with activity buffer, and the pellet and supernatant fractions were evaluated for protein content by SDS-PAGE (12.5%).

#### 2.6.3 Nanoparticle characterization

The Attenuated total reflectance Fourier transform infrared spectroscopy (ATR-FTIR) spectrum was evaluated before (PHA NPs) and after protein functionalization (M711-NPs) to confirm protein incorporation. Briefly, 100 μL of PHA NPs or M711-PHA NPs were centrifuged (13,000 x *g*, 30 min) and freeze-dried. The lyophilized NPs were analyzed in a Perkin–Elmer (Spectrum One) spectrometer equipped with an ATR accessory. Spectra were recorded in the range from 4,000 to 400 cm−1 by 4 scans and with a resolution of 4 cm^−1^.

The PHA NPs formulations were evaluated for their size distribution by Dynamic light scattering (DLS) in terms of hydrodynamic diameter (D_H_) and polydispersity index (PDI) in the activity buffer at 25°C. Measurements were taken in a Malvern Nanosizer NanoZS (Malvern, Worcestershire, United Kingdom) equipped with a 4 mW He-Ne laser (λ = 633 nm), with a scattering angle of 173°. Likewise, the zeta (ζ)-potential was measured by laser Doppler electrophoresis (LDE) using the Zetasizer NanoZS. Each measurement was performed in triplicate. Data are presented as the mean ± standard deviation.

To evaluate the stability of the NPs functionalized with either M711 or just Cpl-711 (*i.e.*, specific binding *versus* unspecific adsorption), equimolar amounts (250 nM) of each protein were incubated with 1 mg of PHA NPs. Then, the pellet fraction after centrifugation (13,000 x *g*, 30 min) containing the loaded NPs, was resuspended in the activity buffer containing Triton X-100 (0.01%, 0.1% or 1%) and incubated for 2 h at RT, which has been reported as an efficient strategy for MinP release from PHA granules (14). The resulting suspensions were centrifuged (13,000 x *g*, 30 min) and evaluated for protein release by SDS-PAGE (12.5%).

Likewise, the eventual release of M711 from the NPs was assessed by incubating M711-NPs for up to 4 h at 37°C in activity buffer, centrifuging them (13,000 x *g*, 30 min) and measuring protein release at the supernatant by spectrophotometry at 280 nm.

### 2.7 Antibacterial activity assays

#### 2.7.1 On resting cell suspensions

Early-exponential phase cultures of *S. pneumoniae* R6 (OD_550_ ≈ 0.3) were centrifuged (2,100 x *g*, 20 min, 4°C), washed, and resuspended in half the initial volume of activity buffer. 180 μL of this resting cell suspension was added to each well in a microtiter plate and then mixed with 20 μL of a protein solution (M711 or Cpl-711) at the desired concentrations or just activity buffer. The plate was incubated at 37°C and OD_550_ was monitored for 1 h with a VersaMax microplate absorbance reader (Molecular Devices, CA, United States). After the incubation time, viable bacterial cell counts were performed by 10-fold serial diluting in activity buffer and plating onto blood agar plates.

Alternatively, the antibacterial activity of NPs formulations was assayed in test tubes by mixing 900 μL of the *S. pneumoniae* P046 resting cells prepared as described in the previous paragraph with 100 μL of i) activity buffer; ii) PHA NPs; iii) M711-loaded PHA NPs (M711-NPs); or iv) equimolar amount of free M711. The tubes were then incubated at 37°C for 4 h and samples were taken every hour to perform viable cell counts. All samples were also observed under the microscope after staining with BacLight LIVE/DEAD kit (Invitrogen, MA, United States) according to the manufacturer’s instructions, in an epifluorescence microscope Leica DM4 B (Wetzlar, Germany) using a ×100 phase-contrast objective, a Lumencor light source and an L5 filter system for green fluorescence observation. Images were analyzed using LAS X software from Leica.

#### 2.7.2 On biofilm

To pre-form *S. pneumoniae* P046 biofilms, this strain was grown in CpH8 medium to OD_550_ ≈ 0.5–0.6 (≈10^8^ CFU mL^−1^) and 100-fold diluted in CpH8 medium. 200 µL aliquots of this dilution were added into each well of 96-well polystyrene plates (Costar 3,595, Corning, NY, United States). These cultures were then statically incubated for 16 h at 37°C to allow the biofilms to form. After that time, the planktonic cells were aspirated and the OD_550_ corresponding to the total growth was measured using a microtiter plate reader (Molecular Devices, CA, United States). To evaluate the biofilm disaggregation activity of the loaded NPs, a crystal violet (CV) quantification assay was used (Domenech et al., 2015). The biofilms were thoroughly washed with activity buffer, and the remaining sessile cells were then incubated for 4 h at 37°C with different treatments in activity buffer: PHA NPs, M711-NPs, 250 nM M711, or just buffer as a control. Following the disaggregation treatment, the planktonic fraction was removed and the remaining biofilm was stained with 50 µL CV (1% w/V) for 15 min, rinsed twice with 200 µL of water, and finally solubilized with 200 µL 95% ethanol. The resulting CV suspension was measured at OD_595_ to evaluate the remaining biofilm. Viable cells were quantified from mechanically disaggregated biofilms in unstained wells.

Alternatively, antibiofilm activity was observed by Confocal Laser Scanning Microscopy (CLSM) (42). In this case, biofilms were grown on glass-bottomed dishes (WillCo-dish; WillCo Wells B.V., Netherlands) using the same conditions as specified before. After incubation, the supernatant was removed, and the treatments were applied in a total volume of 1 mL. CLSM observations were done after LIVE/DEAD BacLight staining with a Leica spectral SP8 confocal microscope and analyzed with the LAS X software. Images represent the x–z projections from XZY stacks at 5-µm intervals planes.

### 2.8 Statistical analysis

Data were obtained from, at least, three independent experiments. One-way ANOVA followed by Tukey’s *post hoc* test was used for multiple comparisons as implemented in GraphPad InStat version 8.0. We indicate significant differences as *p* < 0.05 (*), *p* < 0.01 (**), and *p* < 0.001 (***).

## 3 Results and discussion

### 3.1 Soluble production and antimicrobial characterization of the tagged enzybiotic M711

**FIGURE 1:**
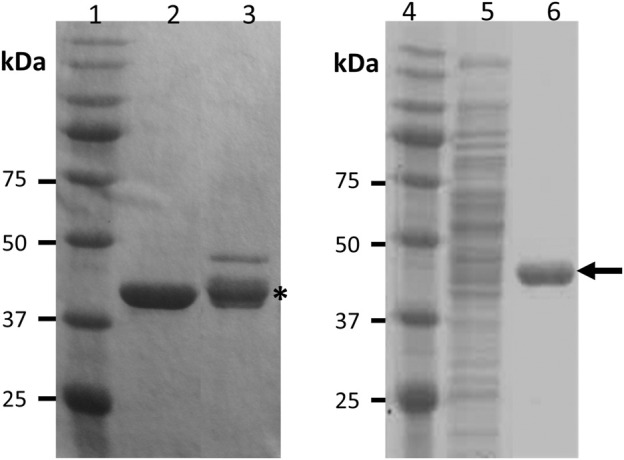
SDS-PAGE images from the M711 purification processes. Lane 1: Molecular weight markers; lane 2: Cpl-711; lane 3: M711 produced in *P. putida* PP00_01 (pFB85) in LB medium. The asterisk indicates a proteolytic product.; lane 4: Molecular weight markers; lane 5: soluble cell extract of *P. putida* PP00_01 (pFB85) grown PHA accumulating conditions; lane 6: isolated granules containing M711 produced in *P. putida* PP00_01 (pFB85) under PHA accumulating conditions. The black arrow indicates the expected size of M711 (44 kDa).

A transcriptional fusion between the PHA affinity tag MinP (Mato et al., 2020) and Cpl-711 (Díez-Martínez et al., 2015) was designed (termed M711) to produce an antipneumococcal protein able to bind to PHA-based materials while remaining functional (Supplementary Figure S1). The new fusion protein (M711) was expressed either in *E. coli* BL21 (DE3) or *P. putida* PP00_01 from pFB85 (Table 1). When expressed from any of the hosts in rich medium (LB), the subsequently purified fractions showed two bands on SDS-PAGE (Figure 1, lane 4). In neither *P. putida* nor *E. coli* did the lower band correspond to the expected size of M711 (43 kDa), but rather to an approximately 40 kDa truncated protein. To identify this product, this lower band was subjected to N-terminal sequencing. The resulting N-terminal amino acid sequence (LVKLE) did not correspond to the expected N-terminus of M711 (MAGKK) but to the C-terminus of the MinP moiety plus the first 3 amino acids of the linker sequence (Supplementary Figure S1B), indicating proteolytic degradation. To prevent this proteolysis, M711 was expressed in *P. putida* PP00_01 under PHA accumulation conditions to associate the MinP-tagged protein onto the granules concomitantly to its synthesis. In this way, the expressed protein would be expected to immediately bind the granules, so then it would remain protected from the potential protease activity. Using this approach, a single band was obtained, and its N-terminal sequence was as expected for M711 minus the processed first methionine (AGKKN, Figure 1, lane 2). This demonstrates direct protein immobilization onto PHA as a powerful tool for the expression of labile proteins.

The protein immobilized onto *P. putida* PHA granules was released by incubating cell extracts with SDS detergent (1.6 mM). In this way, a soluble, full version of M711 was obtained to later be bound to NPs formulated *in vitro*. The released protein was purified by affinity chromatography on a DEAE-Sepharose column (see *Materials and methods*).

**FIGURE 2:**
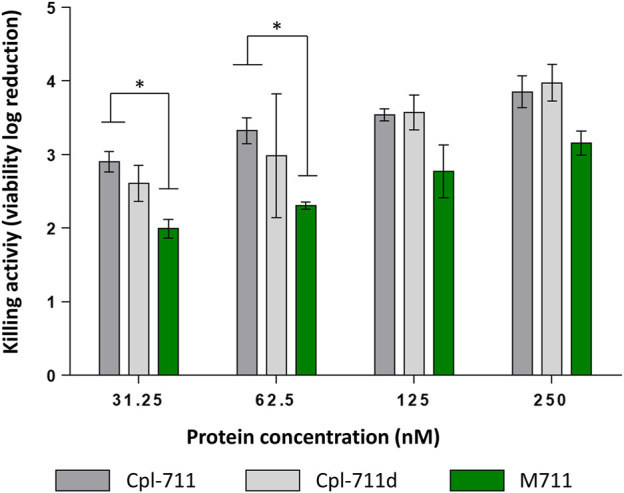
Antimicrobial activity of M711 indicated as logarithmic units of viability reduction regarding an untreated cell suspension. Resting cells were treated with 2-fold dilutions of antimicrobial proteins. Cpl-711 treatments are indicated in dark grey bars; Cpl-711d (light grey bars) means Cpl-711 exposed to SDS during the same time as M711 detached from granules; and cells treated with M711 are indicated in green. An asterisk indicates *p* < 0.05 according to one-way ANOVA followed by Tukey’s post hoc test.

The antibacterial activity of purified M711 was assessed and compared with that of Cpl-711. An additional control of SDS-treated Cpl-711 (Cpl-711d) was also included to exclude the effect of SDS exposure, which could be potentially detrimental to the enzyme. Significant differences were only observed at the two lowest concentrations tested when comparing Cpl-711 with M711 in equimolar concentrations, showing a slight decrease in killing activity (of ∼1 log killing) (Figure 2). No remarkable differences were observed by optical density decrease either (Supplementary Figure S2). This implies that the fused MinP tag does not dramatically hinder the antimicrobial activity and confirms the methodology’s efficiency in producing the tagged enzybiotic M711.

### 3.2 M711-NPs physicochemical characterization

The main infection niche of *S. pneumoniae* is the pneumocytes of the epithelial lung alveoli, below the LS layer (Julio et al., 2022). This means that an inhaled drug conjugate targeting pneumococcal cells, such as the one tested here, should be able to travel through the upper and lower airways to reach the alveoli. Then, the system should be translocated through the layer of LS without losing the loaded drug. The LS is a challenging body barrier consisting of a layer of phospholipids (92%) and hydrophobic proteins (8%) that avoid alveolar collapse during the respiratory mechanics reducing surface tension, as well as plays a role in immune innate defense (Hidalgo et al., 2017).

The ability to reach the lung alveoli is mainly determined by the NP’s size. NPs with D_H_ > 5 μm are usually retained in the upper airways. Those ranging between 1–5 μm reach the lower respiratory tract by deposition, while those with D_H_ ≤ 1 μm arrive at the lung alveoli by Brownian diffusion (Hidalgo et al., 2017).

The second condition, crossing the LS layer, depends on a combination of variables including size, shape, and the chemical nature and surface chemistry of the NPs (Hidalgo et al., 2015). Vehiculization through this surfactant layer needs the formation of an LS corona around the cargo so that it can translocate to the subphase. As LS is mainly composed of lipids, hydrophobic NPs have a better chance of interaction. Also, cationic or neutral NPs are preferred to anionic NPs that can sequester positively charged LS proteins, thus resulting in surfactant dysfunction (Arick et al., 2015). Accordingly, the ability of PHA NPs to translocate through the LS has been experimentally proven (Cañadas et al., 2021). Not only PHA NPs have been demonstrated to translocate through the LS, but also phasins, such as PhaF, from which the MinP tag is derived, have proven to interact with the LS (Mato et al., 2019).

**FIGURE 3:**
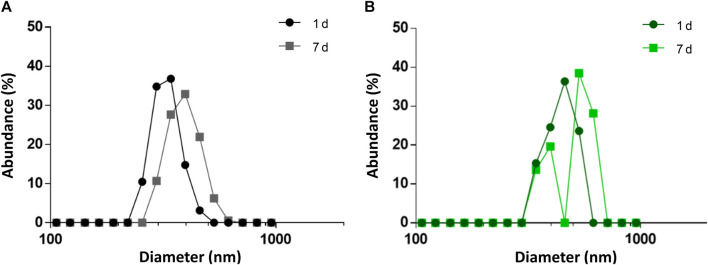
Size distribution of PHA NPs and M711-NPs. **(A)** Size distribution of PHA NPs right after preparation (black circles) and after 7 days of storage at 4°C (grey squares). **(B)** Size distribution of M711-NPS right after preparation (dark green circles), and after 7 days of storage at 4°C (light green squares).

Taking all the above mentioned into account, the PHA NPs formulation hereby tested was designed to meet those requirements. PHA NPs were obtained by nanoprecipitation (see Materials and methods 2.5) without the use of any stabilizer. The presence of surfactants may, on one hand, stabilize NPs systems, but, on the other hand, it interferes with phasin binding (Zhao et al., 2016). Indeed, preliminary results of protein fractionation between supernatant and surfactant-stabilized PHA NPs showed that the detergents intensely interfered with protein binding (Supplementary Figure S3). M711 was then bound to bare PHA NPs by incubating the tagged enzybiotic with the NPs. The incorporation of M711 on the NPS (M711-NPs) was confirmed by ATR-FTIR (Supplementary Figure S4). PHA NPs presented a unimodal distribution with mean D_H_ = 431 ± 14 nm (Figure 3A), which remained stable for a week (final D_H_ = 458 ± 15 nm, also unimodally distributed). Likewise, the size distribution of NPs (M711-NPs) was unimodal but with a larger D_H_ (548 ± 12) (Figure 3B) as measured right after preparation, meeting the size targeted for an eventual alveolar delivery of the NPs. However, the M711-NPs showed a bimodal size distribution after 7 days of storage at 4°C in the activity buffer, with mean sizes at each peak of 392 and 531 nm.

**FIGURE 4:**
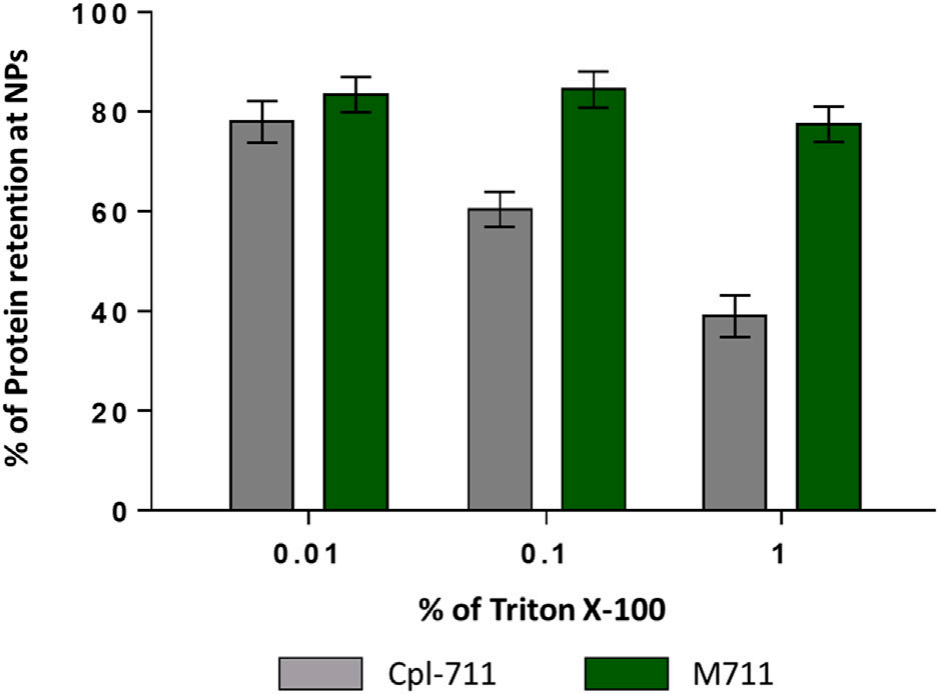
Protein retention at PHA NPs loaded with Cpl-711 (grey bars) or with M711 (green bars). Bars indicate the percentage of the initial protein load that remains immobilized on PHA NPs after elution with increasing concentrations of Triton X-100 as calculated by SDS-PAGE.

This latter result shows that M711-NPs tend to interact with each other and aggregate over time but still maintaining the size requirements for an alveolar delivery MP system. Another interesting remark is the size increment of M711-NPs as compared to that of PHA NPs. Given the dimensions of the crystal structure of Cpl-1 (PDB 2IXU, 67.99 Å × 61.77 Å x 40.08 Å), which is 92% identical in sequence to Cpl-711, a difference in D_H_ of roughly 70 nm would indicate that several layers of protein were formed around the NPs. Although these broad layers of proteins are formed over PHA granules *in vivo* by the oligomerization of phasins through their leucine zipper motives (Tarazona et al., 2019b), these were removed in the design of MinP (Mato et al., 2020). It is however rather well established that the formation of a non-specifically associated protein corona at the NP’s surface is a common phenomenon in protein-crowded solutions and biofluids (Lima et al., 2020). The existence of unspecific binding between the PHA NPs and the Cpl-711 moiety other than that driven by MinP was analyzed by assessing protein binding and release induced by exposure to Triton X-100 by SDS-PAGE. This detergent is known to effectively detach proteins from PHA granules, and it would also somehow mimic the surfactant environment at the LS (Olmeda et al., 2015; Mato et al., 2020). Indeed, the Cpl-711 attached to PHA NPs. However, a concentration-dependent elution was observed for Cpl-711 upon treatment with Triton X-100, while the amount of M711 retained in the NPs remained almost unchanged (at ≈ 80% of total protein) (Figure 4), in agreement with previous results (Mato et al., 2020). Taking this into account, a possible binding scheme could be the soft and hard corona model (García-Álvarez and Vallet-Regí, 2021), in which proteins bound to the PHA NPs via the MinP tag are tightly bound to the NPs while those bound via unspecific interactions of Cpl-711 are loosely attached. The fact that in the presence of surfactants such as Triton X-100 only small amounts of M711 are detached could correlate with smaller cargo losses during an eventual translocation at the LS. Furthermore, the aggregation effect observed when stored in an aqueous solution is likely to be prevented in the presence of LS, where the non-specifically bound protein would be released.

Also related to the colloidal stability of the system, PHA NPs showed a *ζ*-potential of –4.37 ± 0.34 mV, which was further reduced upon functionalization down to –7.46 ± 0.58 mV. Particulate systems are generally accepted to be stable with a *ζ*-potential of ±30 mV (Pochapski et al., 2021). In this case, a balance between the charge needed for stabilizing the system for long-term storage and the charge desired for facilitated transport through the LS (neutral or cationic) should be addressed. Our values are rather neutral, which was nevertheless expected given the absence of charged moieties in PHA (Abid et al., 2016; Lee et al., 2022).

### 3.3 M711-NPs antimicrobial characterization

After proving the suitable physicochemical properties of M711-NPs, the antibacterial activity of the system was evaluated. Indeed, the immobilization of enzymes is very challenging, due to the need of maintaining the three-dimensional structure of the protein and its orientation to keep the biological activity. Because *S. pneumoniae* presents spontaneous autolysis when reaching the stationary growth phase, an autolysin-devoid pneumococcal strain P046 (Moscoso et al., 2006) was used to assess the antibacterial activity of the M711-NPs over time. Determination of the Minimal inhibitory concentration could not be performed due to the turbidity provided by the NPs. Thus, we directly assessed the M711-NPs with *S. pneumoniae* resting cells.

**FIGURE 5:**
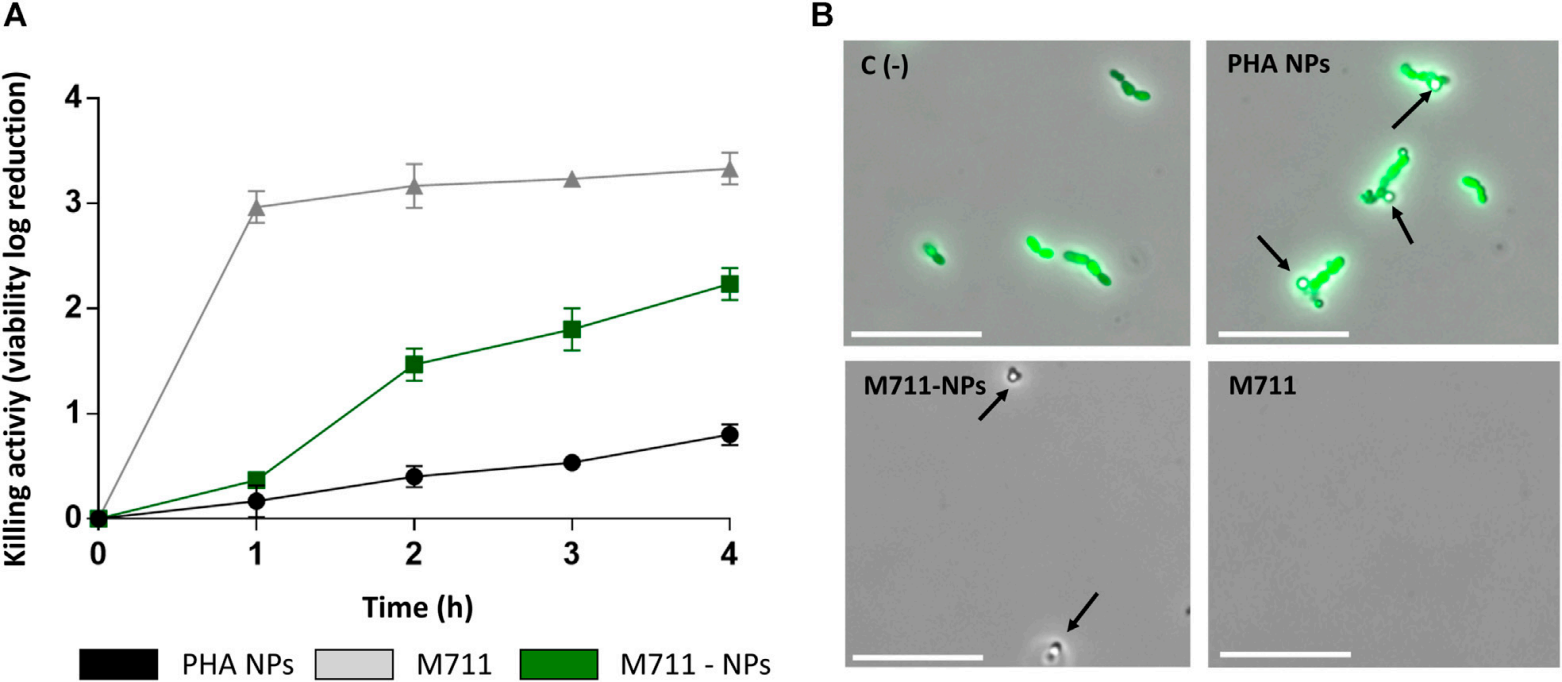
Antibacterial activity of M711-NPs against *S. pneumoniae* P046. **(A)** Time-killing profile of logarithmic units of viability reduction of free M711 (grey line), M711-NPs (green line), or blank PHA NPs (black line). **(B)** Fluorescent microscopy images of resting cell suspensions after 4 h of incubation with each treatment. No cell viability alteration was observed in untreated cells or cells treated with PHA NPs (green cells indicate metabolically active cells). Meanwhile, cells treated with M711-NPs and free M711 were strongly affected in viability, and only the remaining M711-NPs (indicated by black arrows) could be seen under the microscope. Scale bars are 10 µm.

In a killing activity assay prolonged for 4 h (Figure 5) the free M711 outperformed both blank NPs and M711-NPs, with a killing curve that peaked already after 1 h. PHA NPs had a little killing effect on their own, while M711-NPs with a protein concentration equivalent to that of the free M711 treatment displayed a time-dependent sustained antibacterial activity. M711-NPs reached a killing activity of 2.2 units (Figure 5A), meaning that ≈99% of the *S. pneumoniae* cells were eliminated. Fluorescence microscopy results (Figure 5B) showed the aggregation of PHA-NPs around the cells without producing any apparent alteration in cell viability (green cells, Figure 5B). Conversely, after 4 h of incubation with M711-NPs, no cells could be observed, but only the remaining M711-NPs, which further confirmed bacterial cell lysis.

The MinP tag had been previously used to functionalize PHA granules *in vivo* leading to active immobilized enzymes (Mato et al., 2020). However, this is the first time this tag has been used to immobilize an enzyme *in vitro* while preserving its activity. While the conversion of small chemicals by enzymes immobilized on PHA granules can occur at the functionalized granule itself, the antibacterial drug delivery formulations usually imply the release of the active molecule. However, our results strongly suggest that the antimicrobial activity was due to the protein attached to the particle since no protein was detected by spectrophotometric measurements at 280 nm on the supernatant of M711-NPs incubated for up to 4 h at 37°C. The lack of release would also explain the observed lag in the activity kinetics (Figure 5A). Different causes could be argued for the kinetic retardation assuming the lack of cargo release, for example i) the steric hindrances partially blocking the access of immobilized enzymes to their substrate; ii) a lesser number of target bacteria reachable by clustered (immobilized) enzybiotics regarding an equimolar concentration of free enzymes, able to diffuse as individual molecules; iii) as multiple layers are probably formed as suggested by the increment in particle size, probably only the outermost layer displays an effective antimicrobial activity. Indeed, this behavior has been observed upon irreversible immobilization of enzybiotics. Recently, a study comparing covalent binding and simple adsorption of the antistaphylococcal enzybiotic Auresine*Plus* onto PLGA/chitosan fibers concluded lower and slower death rates for the permanently immobilized material than in the case of the enzybiotic-releasing material (Urbanek et al., 2021). However, a sustained activity due to immobilized, more stable enzybiotic could be preferred to a fast release in a clinical setting (Gao et al., 2011). Indeed, a major advantage of irreversible immobilization may be the protection of the enzyme against proteolytic activities (*e.g.* as observed in Figure 1). In contrast, in a self-releasing system, the released enzybiotic would still be susceptible to such degradation and would probably have a short residence time within the body.

**FIGURE 6:**
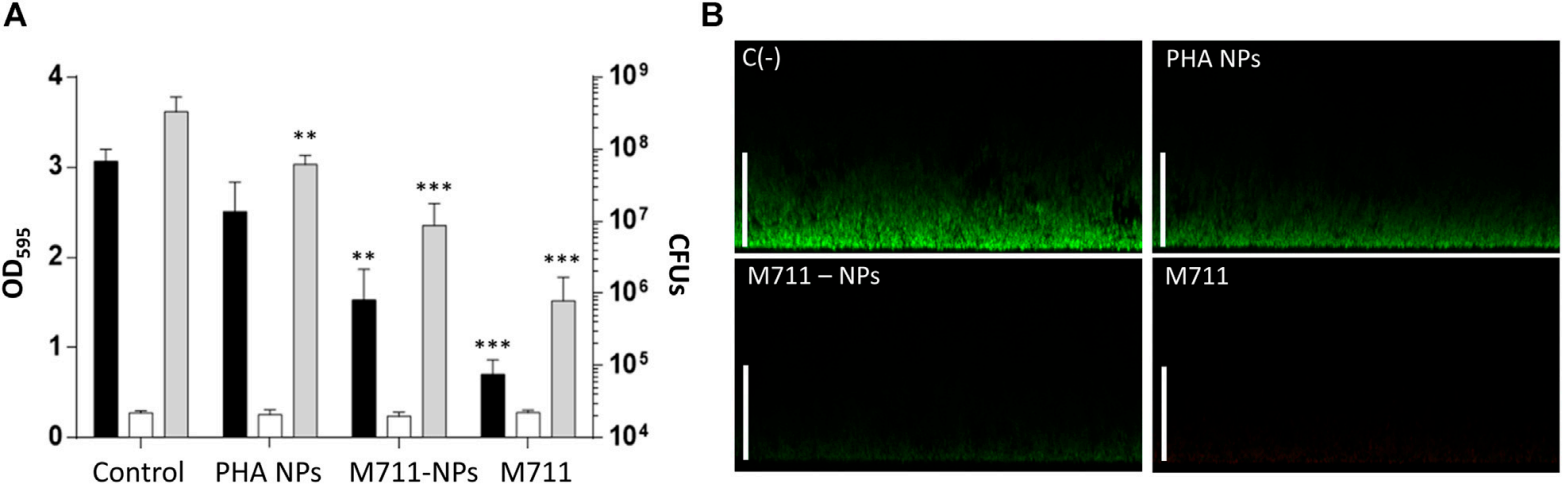
Antibiofilm activity of M711-NPS system. **(A)** Biofilm disaggregation and viable cells count from mature biofilms treated for 4 h with activity buffer, PHA NPs, M711-NPs, or free M711 in equimolar amounts (250 nM). Black bars indicate the remaining biofilm as assessed by CV staining, white bars indicate growth controls of the planktonic cell from the biofilm well, and grey bars indicate the number of viable cells in each biofilm per well. Asterisks indicate different levels of signification following a one-way ANOVA with a Tuckey post hoc test comparing each treatment with the control. (**) *p* < 0.01; (***) *p* < 0.001. **(B)** CLSM of untreated and treated biofilms with PHA NPs, M711-NPs, or free M711 for 4 h. Images are the views of the maximal projection on the x-z plane. Scale bars indicate 25 µm.

Because the activity of the M711-NPs is thought to need the direct contact between the loaded NPs and bacteria, the antibacterial activity of the NPs was assessed on a solid substrate (biofilm). Due to this sessile lifestyle, the contact between the NPs and the *S. pneumoniae* cells will be more intimate than that on cell suspensions. Importantly, *S. pneumoniae* is able to form stable biofilms associated with up to 80% of chronic infections and persistent conditions (Wolcott and Ehrlich, 2008). The disaggregation of the biofilms was 13% for PHA NPs, 50% for the M711-NPs, and 77% for the free M711 treatment, with respect to an untreated control biofilm (Figure 6A), as assessed by CV staining. These results were further confirmed by CLSM, where observations indicated that the thickness of the biofilm was reduced from ≈25 µm to ≈15 μm, ≈5 µm or up to unmeasurable levels, when treated with PHA NPs, M711-NPs or free M711, respectively (Figure 6B). In terms of antibacterial activity, the viability reduction of the biofilms was significantly decreased by 0.5, 1.2, and 2.3 logarithmic units for PHA-NPs, M711-NPs, and free M711, respectively (Figure 6A). Altogether, these results indicate that M711-NPs display pneumococcus biofilm disaggregation activity despite the absence of enzybiotic release (Figure 5). Moreover, the reduced specific activity of the immobilized enzybiotic, is compensated by other functional advantages of immobilization, such as an increase of *in vivo* half-life regarding the administration of the free enzyme or by an enhanced ability to be vehiculized through the LS, as demonstrated for PHA NPs, in a possible M711-NPs inhaled formulation.

## 4 Conclusion

In this work, we proposed tag-mediated immobilization of enzybiotics as a method for the antimicrobial functionalization of PHA-based materials. We first achieved the *in vivo* immobilization of the fusion protein M711 onto PHA granules, which prevented the proteolytic degradation observed for the soluble production of the protein in two different heterologous hosts. This demonstrates *in vivo* PHA immobilization as a tool for the production of labile proteins. The protein purified from the granules through detergent washing was able to bind again onto preformed PHA NPs *in vitro*, and the functionalized NPs displayed antibacterial activity against both sessile and planktonic *S. pneumoniae* cells. Although the specific antimicrobial activity of M711-NPs was slightly reduced as compared with equimolar amounts of the free enzyme, this is expected to be compensated by other therapeutical properties of the NPs formulation, such as a prolonged half-life or the enhanced translocation through body barriers (*e.g*., the LS, as described for PHA NPs). These advantages remain however to be explored in future studies.

## Data Availability

The original contributions presented in the study are included in the article/Supplementary Material, further inquiries can be directed to the corresponding author.
