# Supplementary material for: Enzybiotic-mediated antimicrobial functionalization of polyhydroxyalkanoates
Source: Front Bioeng Biotechnol. 2023 Jun 28;11:1220336. doi: 10.3389/fbioe.2023.1220336 (PMC10336440; doi:10.3389/fbioe.2023.1220336)
Supplement: Supplementary file 1 [file DataSheet1.DOCX]

Supplementary Material

Enzybiotic-mediated antimicrobial functionalization of polyhydroxyalkanoates

Francisco G. Blanco^1,2^, Roberto Vázquez^3,†^, Ana María Hernández-Arriaga^1,2^, Pedro García^3,*^, and M. Auxiliadora Prieto^1,2^

^1^ Polymer Biotechnology Group, Microbial & Plant Biotechnology Department, Margarita Salas Center for Biological Research (CIB– CSIC), Madrid, Spain

^2^ Interdisciplinary Platform of Sustainable Plastics towards a Circular Economy, Spanish National Research Council (SusPlast-CSIC), Madrid, Spain

^3^ Protein Engineering Against Antibiotic Resistance Group, Microbial & Plant Biotechnology Department, Margarita Salas Center for Biological Research (CIB-CSIC), Madrid, Spain

^†^ Current address: Laboratory of Applied Biotechnology. Department of Biotechnology. Ghent University. Ghent, Belgium

*** Correspondence:**
M. Auxiliadora Prieto: [auxi@cib.csic.es](mailto:auxi@cib.csic.es) Pedro García: [pgarcia@cib.csic.es](mailto:pgarcia@cib.csic.es)

**
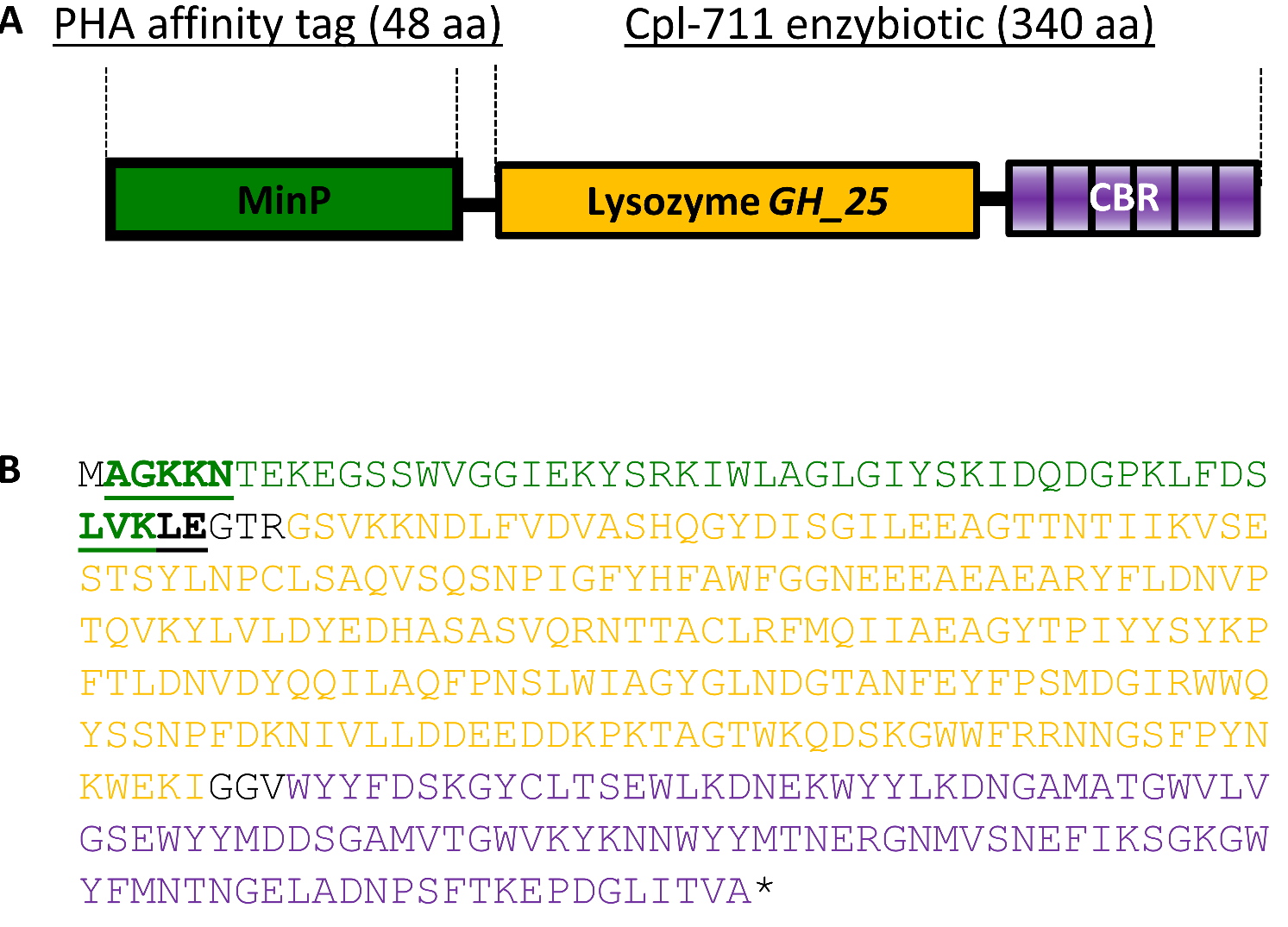
**

**Figure S1. M711 chimeric construction.** A) Schematic representation of the architecture of M711. The functional domains are indicated in green for the PHA affinity tag (MinP); in orange for the catalytic lysozyme domain; and in purple for the choline-binding repeats (CBR) or cell wall binding domain. B) Amino acid sequence of M711. Each functional domain is highlighted in the same color as in the upper panel. Underlined sequences indicate the sequences obtained by N-terminal sequencing.

**
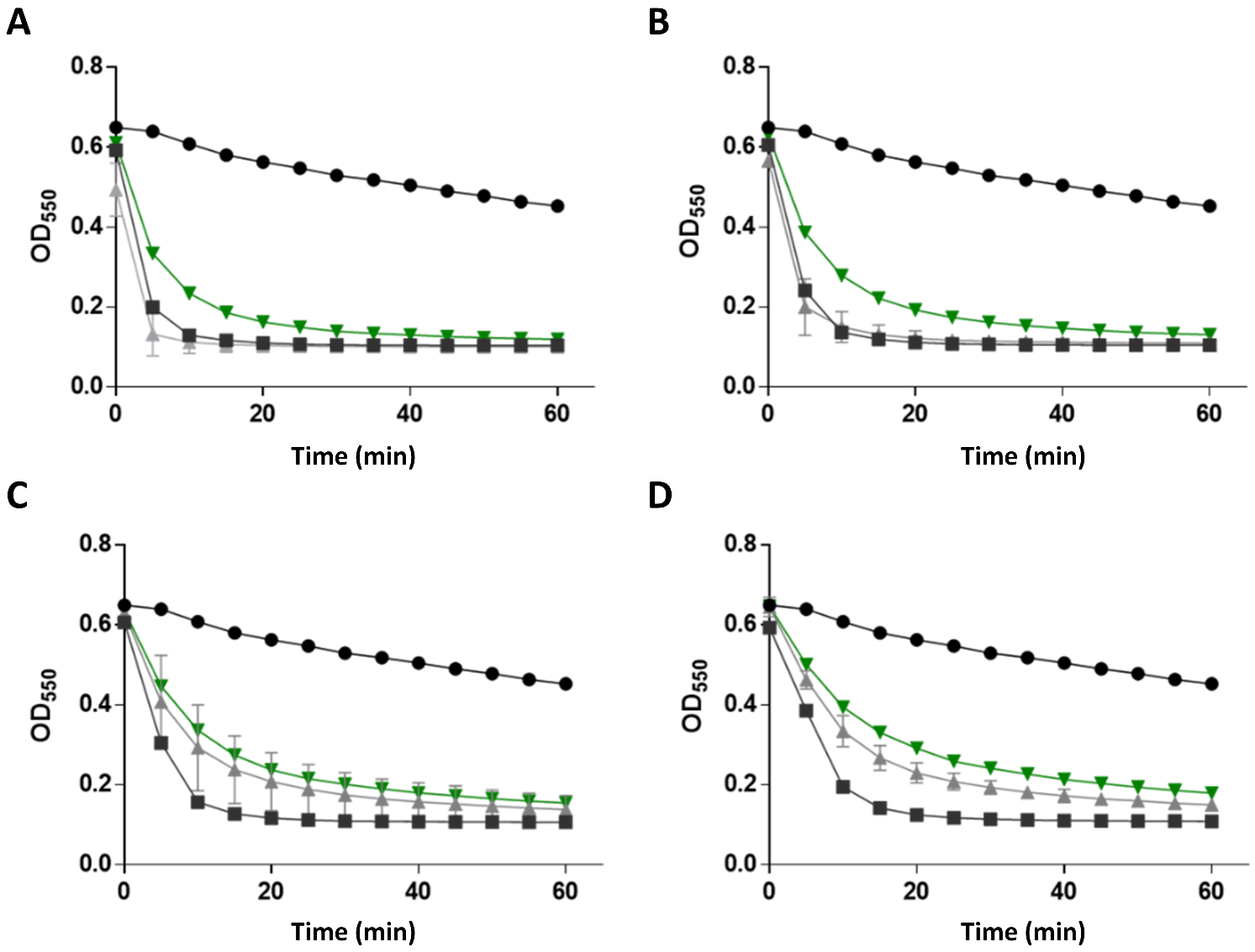
**

**Figure S2. Optical density decrease at 550 nm with different antimicrobial proteins at 250 nM (A), 125 nM (B), 62.5 nM (C), or 31.25 nM (D).** Black circles indicate a negative control of untreated resting cells; dark grey squares indicate cultures treated with Cpl-711; light grey triangles indicate a control of SDS-treated Cpl-711 (Cpl-711d); and green triangles indicate M711 treated cultures.

**
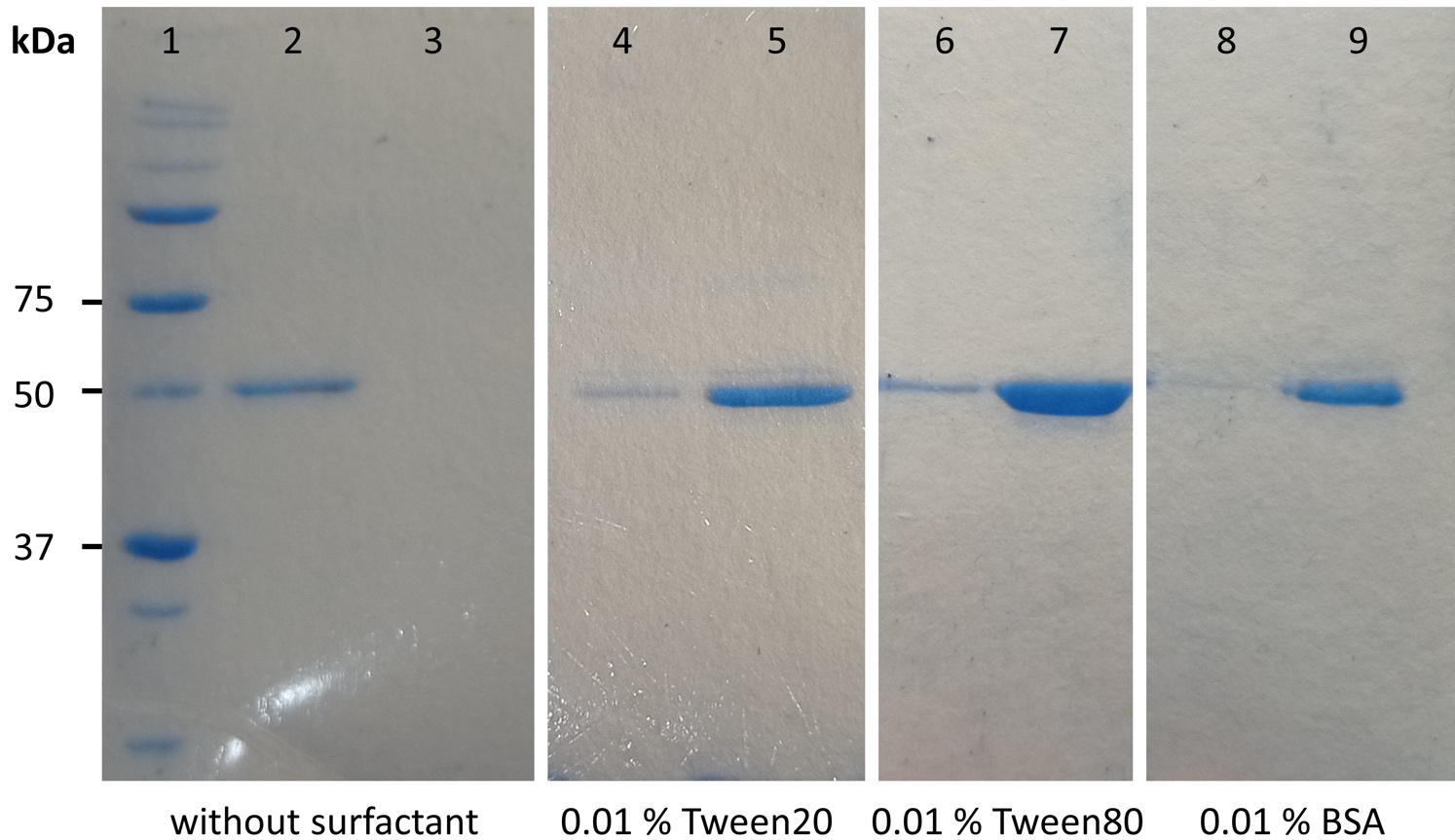
**

**Figure S3. M711 distribution between pellet and supernatant fractions after centrifugation, as assessed on 12.5 % SDS-PAGE for PHA NPs stabilized with different surfactants.** Lane 1: Molecular weight markers; lanes 2,4,6,8: pellet fractions after centrifugation containing the NPs (immobilized fraction); lanes 3,5,7,9: supernatant fractions of the NPs after centrifugation (unbound fraction). Surfactants on the NPs are indicated. BSA stands for bovine serum albumin.


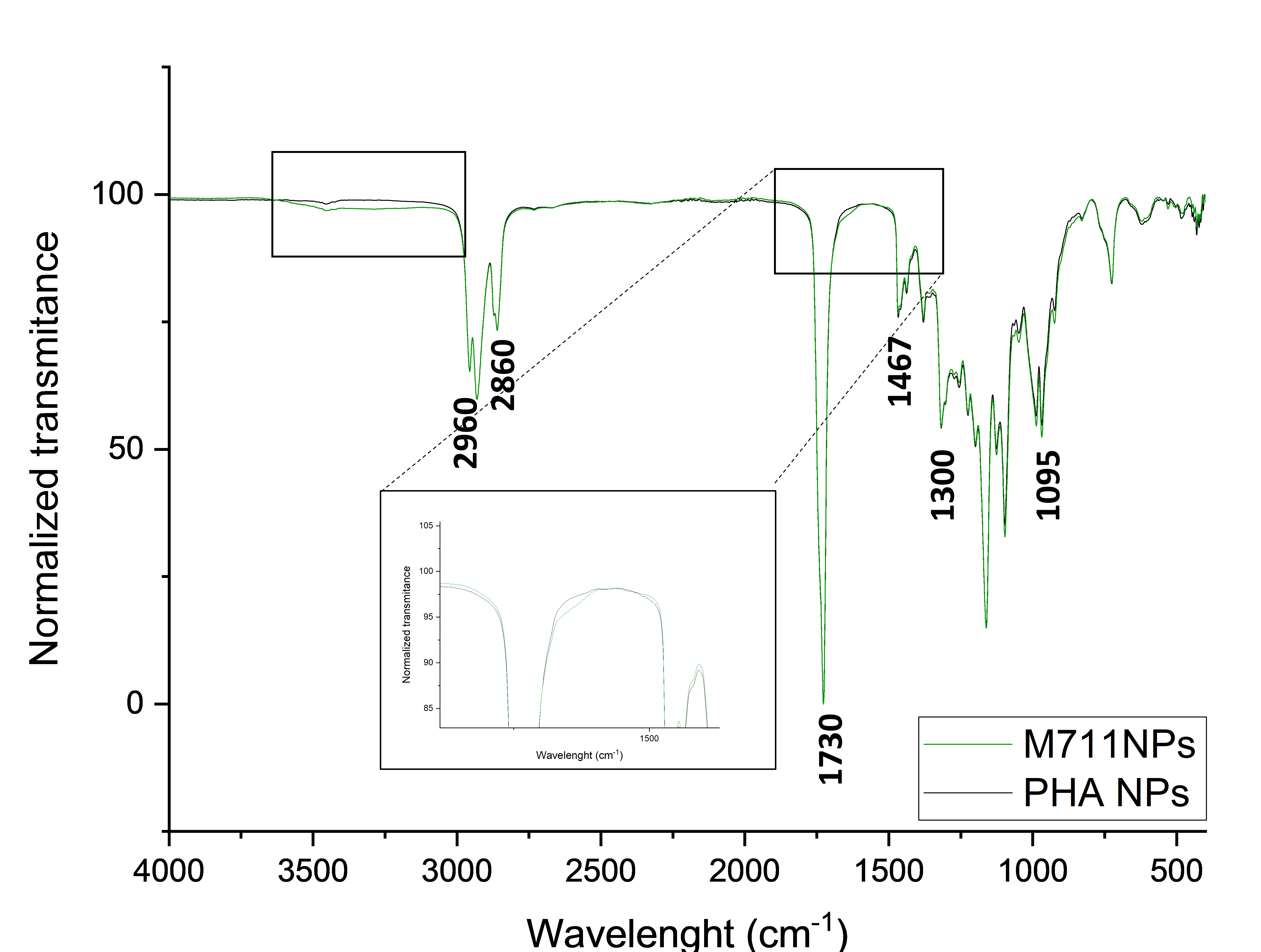
**Figure S4. ATR-FTIR spectra of PHA NPs and M711- NPs.**

The ATR-FTIR spectra of PHA NPs showed the typical spectrum of PHOH. The stretching of the C=O double bond was detected as a high-intensity peak at 1730 cm^-1^. The CH stretching of the aliphatic backbone produced several peaks between 2860-2960 cm^-1^. The stretching of the CO bond in the ester group was detected as medium-intensity peaks between 1095-1300 cm^-1^. Finally, the CH_2_ bending of the side chains appeared as peaks in the range of 1300-1467 cm^-1^. (1)

All of these peaks were present in the functionalized NPs (M711). The main difference between the bare PHA NPs and the M711-NPs ATR-FTIR spectra is found in the region between entre 1650-1640 cm^-1^, which is due to the amide I group, showing the presence of the protein on the surface of the NPs. The primary cause of the amide I vibration, which occurs at approximately 1650 cm^−1^, is the stretching of the C=O double bond, although it also includes slight contributions from the out-of-phase CN stretching vibration, the CCN deformation, and the NH in-plane bend (2). In the case of the NPs used in this study, the intensity of this band is low due to the low relative amount of protein regarding the PHA carrier 0.1% (w/w). Furthermore, the presence of the protein on the modified NPs (M711-NPs) is also observed by the amide A band due to the NH stretching vibration between 3310 and 3270 cm^−1^ (2).

1. Shamala TR et al., Production and characterization of bacterial polyhydroxyalkanoate copolymers and evaluation of their blends by fourier transform infrared spectroscopy and scanning electron microscopy. Indian J Microbiol. 2009;49(3):251–8.

2. Barth A. Infrared spectroscopy of proteins. Biochim Biophys Acta BBA - Bioenerg. 2007;1767(9):1073–101.
